# Simulating a patient's fall as a means to improve routine communication: Joint training for nursing and fifth-year medical students

**DOI:** 10.3205/zma001018

**Published:** 2016-04-29

**Authors:** Markus Flentje, Thomas Müßel, Bettina Henzel, Jan-Peter Jantzen

**Affiliations:** 1 Medizinische Hochschule Hannover, Klinik für Anästhesiologie und Intensivmedizin, Hannover, Germany; 2 Klinikum Region Hannover, Ausbildungszentrum, Hannover, Germany; 3 Klinikum Region Hannover, KRH Klinikum Nordstadt, Klinik für Anästhesiologie, Intensivmedizin und Schmerztherapie, Hannover, Germany

**Keywords:** Interprofessional collaboration, patient fall, simulation training, debriefing, communication

## Abstract

**Background: **Physicians and nursing staff interact as a team on a daily basis in hospital settings. However, both educational paths offer few opportunities to establish contact with the other professional group. Neither professional group can practice its later role with the other group in a “safe” learning environment. Routine interprofessional collaboration is described as being in need of great improvement and carries with it the potential for conflict. To improve interprofessional communication and task management, a simulation-based emergency training session for nursing students and fifth-year medical students was developed at the KRH Klinikum Nordstadt in Hanover, Germany. As a pilot project, the course was held twice in the form of a one-day session with ten nursing and four medical students.

**Project: **Using the example of a patient’s fall, course participants were able to observe and actively treat multiple simulated patients. Following each simulation the trainer conducted a comprehensive debriefing. The course was then evaluated using a questionnaire.

**Results: **The evaluation of the team training showed a high level of acceptance among the two participating professional groups. On a scale of 1 (hardly applicable) to 5 (strongly applicable), the course was given a 4 by both professional groups for its relevance to daily work. In the open-ended written responses praise was specifically given for the opportunity to learn how to switch perspectives as a result of the simulation exercises.

**Conclusion:** A common emergency on the hospital ward offers a good opportunity to establish and practice interprofessional team skills. With the knowledge gained about communication and the ability to change viewpoints, participants are able to improve their team skills. Participants demonstrated a high degree of acceptance for the training program.

## Zusammenfassung

**Hintergrund: **Pflegende und Ärzte agieren im Krankenhaus täglich als Team. In beiden Ausbildungsgängen sind allerdings nur wenige Berührungspunkte mit der anderen Berufsgruppe vorgesehen. Keiner der Berufsgruppen kann seine spätere Rolle mit seinem Gegenüber im geschützten Rahmen einüben. Die interprofessionelle Zusammenarbeit im Berufsalltag wird als stark verbesserungswürdig beschrieben und beinhaltet Konfliktpotential. Zur Verbesserung der interprofessionellen Kommunikation und des Aufgabenmanagements wurde am KRH Klinikum Nordstadt Hannover ein simulatorbasiertes Notfalltraining für Krankenpflegeschüler und PJ-Studenten entwickelt. Der Kurs wurde als Pilotprojekt in Form eines Tageskurses zweimal mit jeweils zehn pflegerischen und vier ärztlichen Teilnehmern durchgeführt.

**Projekt: **Anhand der Beispielsituation „Sturz eines Patienten“ konnten die Kursteilnehmer sowohl als Zuschauer, als auch als Teilnehmer mehrere Simulationspatienten behandeln. Nach jeder Simulation wurde durch die Trainer ein ausführliches Debriefing durchgeführt. Die Veranstaltung wurde anschließend mittels eines Fragebogens evaluiert.

**Ergebnisse: **Die Evaluation des Teamtrainings zeigt eine hohe Akzeptanz bei den beteiligten Berufsgruppen. Auf einer Skala von 1 (trifft kaum zu) bis 5 (trifft stark zu) wurde der Kurs von beiden Berufsgruppen mit 4 als relevant für die tägliche Arbeit bewertet. In den Freitexten wurde ausdrücklich der mögliche Perspektivwechsel durch die Simulation gelobt.

**Schlussfolgerung: **Notfallsituation aus dem Stationsalltag stellen eine gute Möglichkeit dar, interprofessionelles Teamtraining zu etablieren. Mit dem Erkenntnisgewinn im Bereich Kommunikation und Perspektivwechsel erhöhen die Teilnehmer Ihre Kompetenz im Bereich Teamarbeit. Das Training erfuhr bei allen Beteiligten eine hohe Akzeptanz.

## 1. Introduction

The goal in educating nurses and physicians is the professional and ensured provision of health care to patients in medical institutions. This goal is not always achieved in the American healthcare system, as indicated by the study “To Err is Human”; medical errors accounted for 44,000 deaths in a year [1]. Another study identified errors in 11% of cases in British hospitals as being human in nature and avoidable in 46% [2]. Teams bearing high responsibility for the life and health of others and whose irreversible actions are taken under time pressure are also referred to as “high responsibility teams” [3]. In addition to workers in the healthcare system, those working in law enforcement and aviation engage in high risk occupations. In the field of aviation, crisis management seminars, in particular, have been developed as a means of crew resource management to account for and do justice to the human factors in these systems. Attending such training is required both during formal training and later during the professional career. Effective teamwork under extreme pressure is the goal of this training. Communication and quality teamwork belong to the key elements defined as promoting successful teamwork in the field of medicine. These skills should be practiced primarily in training sessions with interprofessional simulation [4].

In the courses offered at the simulation center of the Klinikum Nordstadt strategies for improving collaboration are covered, in addition to the medical topics. The abilities needed to master a situation are divided into technical and non-technical skills [5]. In the medical setting these seminars are also called crisis resource management. To be in a better position to evaluation critical situations and, above all, the non-technical skills, the guiding principles put forth by of Gaba and Rall (see attachment: text box 3 ) are applied to the debriefing sessions.

Currently, nurses and physicians are educated separately. A nursing internship during the pre-clinical phase of formal medical education offers a brief opportunity for contact between the two groups. Due to the lack of practical professional experience students are unable at this point in their education to assess and consider the skills and competencies necessary for successful interprofessional teamwork. The National Competency-based Catalogue of Learning Objectives for Undergraduate Medical Study was not yet available when this project began [http://www.nklm.de].

Within this context, it is hardly surprising that many online discussions and blogs take place about the importance and meaning of the nursing internship, including very emotional posts about “physician-nurse conflicts”. While writing this article, an internet search using these three terms yielded 383,000 hits using a popular search engine. Communication problems, lack of respect for others, and unclear definitions of skills and responsibilities are cited as the roots of these conflicts [6]. A final report of a study on interprofessional communication in hospitals asserts that cooperation between physicians and nursing staff is in need of great improvement [7].

It is to be feared that, under growing economic pressures and higher workloads, potentials for conflict and poor communication will only increase. This indicates much more than simply a cosmetic flaw in the quality of interprofessional collaboration; connections between communication, hierarchy, team management and the quality of patient safety and health care are seen [4]. For this reason, the hypothesis was posed that a joint seminar for both groups prior to completing formal education will be evaluated positively.

## 2. Project description

The FIPPS simulation center^1^ at the Klinikum Nordstadt is part of the Anesthesiology, Intensive Medicine and Pain Therapy Clinic and has, since 2009, developed several simulation courses for the Anesthesiology Department addressing critical hospital situations and patient safety [8]. When training for specific situations, such as an emergency cesarean [9], airway management orresuscitation, the professional groups affected in real situations always participated. In connection with this, organizers received positive feedback explicitly concerning the cooperation between physicians and nurses. In particular, improvement of communication between the professional groups was repeatedly identified as a worthwhile reason for attending the training courses. Based on this positive experience with team training, questions arose as to why joint training takes place only after the completion of formal training, and would it not make more sense to make this possible sooner. Team training for nursing and fifth-year medical students was then initiated as a pilot project. The aim of this project was to see if, at this point in formal education, imparting information about crisis management is perceived as important. The participants were supposed to learn, in different simulated settings as team members and observers, how communication, resource management and problem-solving skills can contribute to successful mastery of a critical hospital situation. The professional skills to be acquired were defined in advance as the learning objectives for a critical situation (see attachment: text box 1 ). The competency model for the Clinic’s in-house training center was used based on the guidelines for nursing put forth by Lower Saxony’s Ministry of Culture [10]. The main focus was not on methodological competency, since, in the opinion of the authors, this receives sufficient attention in the formal education programs. More specifically, the abilities described under the heading of “social skills” meet the criteria that are applied in connection with patient safety and the provision of health care.

### Simulated scenarios – falling

The clearest indication for training ward personnel jointly is resuscitation. There are established training procedures and materials for this purpose; however, the organizers felt resuscitation was not suitable as the topic of this training course. The reasons were that, in the case of reanimation, the roles and responsibilities of the different professions are clearly defined, the medical procedures are clearly set down in guidelines, and rules govern the transfer of a patient to an intensive care unit. The decisions needing to be met are too clear for training communication skills and the ability to handle and avert conflict in routine work. In the course of searching for an appropriate situation that met relevant and realistic criteria from the participants’ perspective, the organizers agreed upon a patient’s fall as an “event” that could provide a general training scenario. In the larger scenario, different pathophysiologies and therapies can be explored so that extensive communication must take place between the participants (see attachment: text box 2 for the range of scenarios ).

#### Course sequence

The training course was designed to be a one-day course of six units (unit=45 minutes). A total of two nursing students and eight fifth-year medical students participated in two training days. The course was integrated into the nursing program, specifically into the module for organizing and arranging nursing care. This involved the curriculum of the training center. The fifth-year medical students were active at the KRH Klinikum Nordstadt and signed up voluntarily to take the course. Attendance was voluntary for all participants, who could have dropped out at any time. Any expenses incurred by those participating were not reimbursed. The written evaluations were submitted anonymously. The training units took place in a simulated hospital room. Participants who were not directly involved in the simulation had the opportunity to observe and take notes. No video recording was made. Nearly all simulations were carried out on the patient simulator Kelly^®^ (Laerdal Medical, Norway), which displays very realistic anatomy and simulates different vital functions. Using a remote control it is possible for the operator to speak through the doll. Only the simulation of dementia was done by a actor. The course was led by a certified CRM trainer and physician and two instructors from the training center.

Since crisis management is not taught in either nursing training or medical education, the day-long course began with a unit introducing the topic. During this introductory unit, reservations were identified prior to the simulation, including the fear of having to perform “onstage”. The role of the trainers to moderate and provide guidance for crisis management was again expressly stated. The six core elements of debriefing outlined by the Boston Center of Medical Education [11] were shared as the basis for successful training.

During the second unit, the topic of conflicts between the professional groups was addressed. In the course discussion, example situations of conflict in the clinical setting were examined and analyzed from different points of view. Students’ personal experiences were also included. The unit’s objective was learning to recognize potentials for conflict and accept them as having an influence on routine communication.

The third theoretical unit covered the pathophysiology of a fall brought on by syncope or stumbling, with the possible resulting injuries. A strategy for verifying vital functions, performing physical examination, and addressing necessary further care of the patient was presented to the students. Making all students aware of the procedure for providing emergency care is a goal. All three theoretical units are meant to place the participants on the same level concerning theoretical knowledge of the skills necessary to provide emergency care.

**Figure 1:**
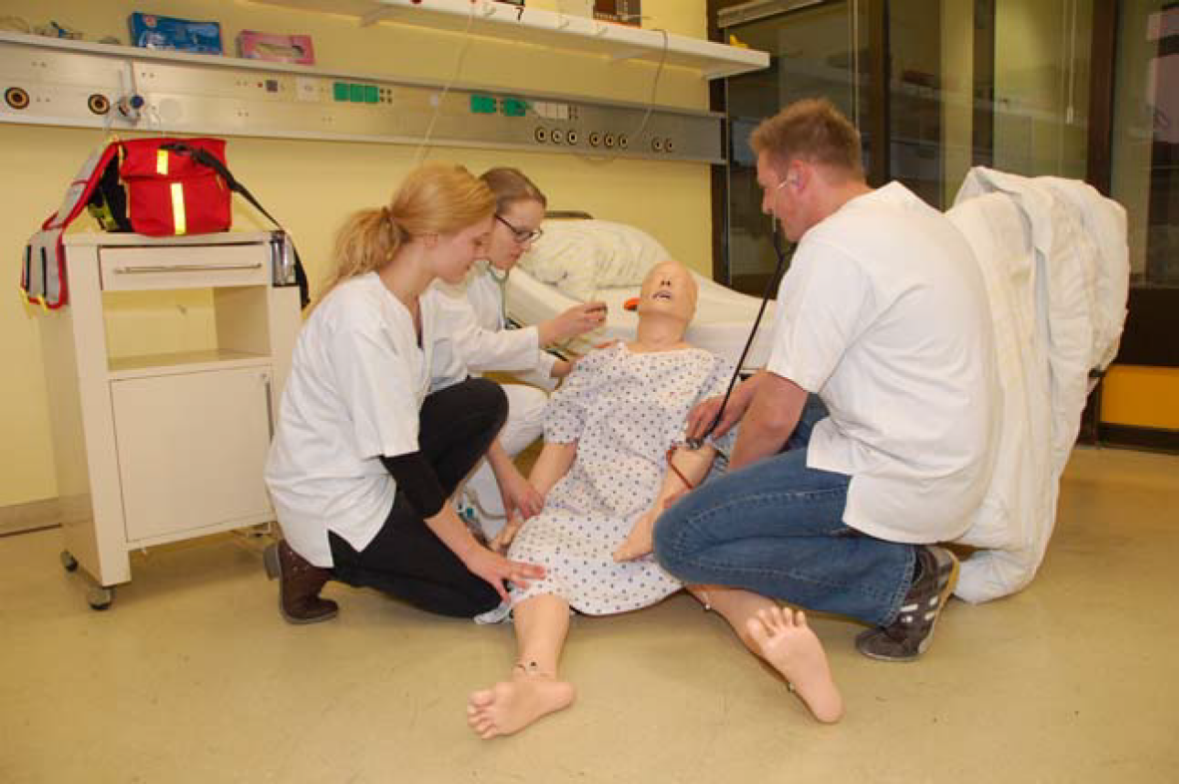
A fifth-year medical student (in background) and two nursing students assist the fallen “patient”.

The enacted scenarios basically consist of three phases. During the first phase the participants must assess the acute risk to the patient. To accomplish this, the vital functions are taken and a full-body examination performed (see Figure 1 (Fig. 1)). In the second phase, treatment according to priority is expected. In the case of hypoglycemia these measures should focus on placing the patient on his or her side if unconscious and administering glucose intravenously. If a fracture is present and the patient is stable, treatment for pain should take place through strategically positioning the patient and pharmacotherapy. In the third phase decisions about further care, diagnostics, and whether or not to transfer the patient to the ICU must be made.

Following each simulation there was a 15-minute debriefing, during which the positive aspects were emphasized by the trainers. Afterwards, the teams were responsible for suggesting improvements regarding their simulated scenario. Particular value was placed on the team members all being simultaneously aware of the relevant suspected diagnoses, therapies and priorities when giving the emergency care. At the end the observers were invited to share their views. Structuring the session in this manner prevented participants from feeling criticized by the outside viewers right from the start of the debriefing. The debriefing concept was pointed out to participants as a good example for structuring follow-up discussions in the hospital setting.

At the close of each day, feedback was gathered from the participants (flash method) in order to implement immediate changes to the course if needed.

Upon conclusion of the training course a written evaluation was carried out using a questionnaire. Six statements were evaluated on a five-point scale of 1 (hardly applicable) to 5 (strongly applicable). The questions were drafted by the trainers with the goal of assessing the cooperation among them in regard to shared cognitive models (questions 1 and 2), the general sense of team among physicians and nurses on the ward, along with participant experiences during training (question 3 and 4), and identifying the importance of the simulated training to education (questions 5 and 6). Questions 1 and 2 cover the competency objective of “adequate communication”; questions 3 and 4 address “successful teamwork”, and questions 5 and 6 the evaluation of the “simulation” as a teaching method. Analysis and description is done with mean values and standard deviation using Excel^©^ (Microsoft, Redmond, WA, USA). In addition, participants had the opportunity to share their evaluations of the course in essay-like written responses.

## 3. Results

**Figure 2:**
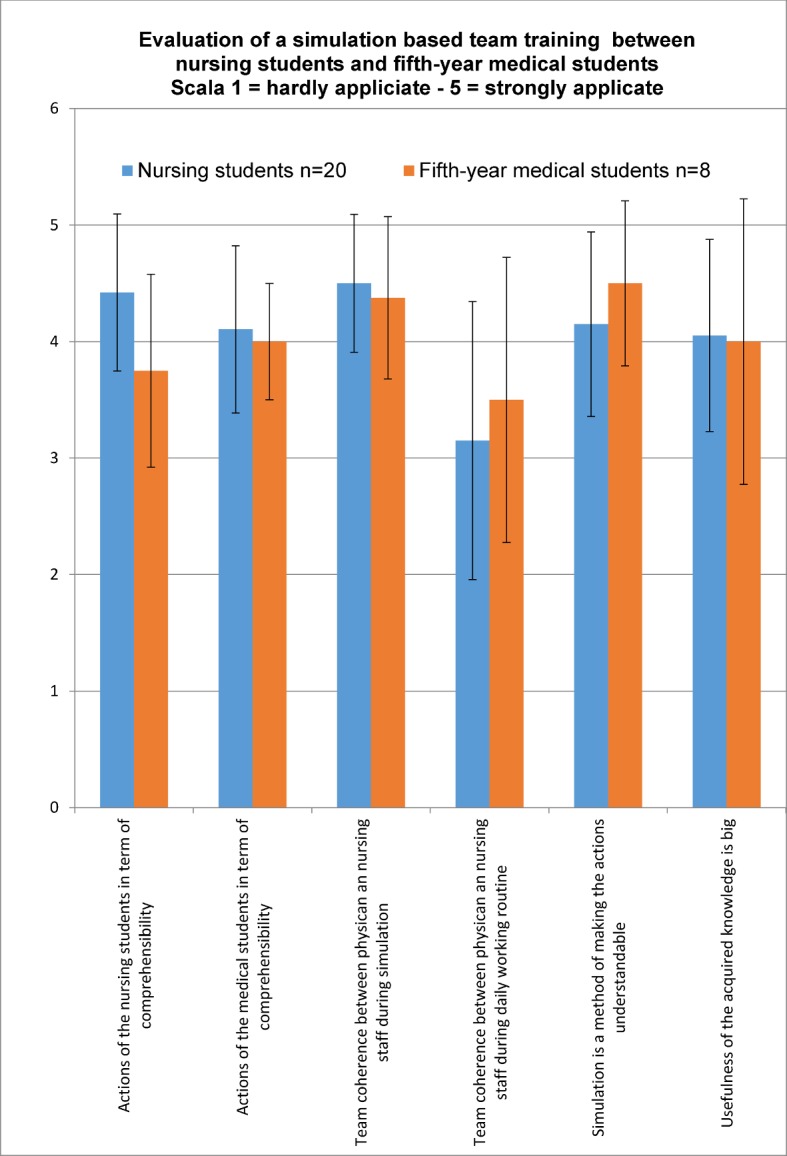
Course evaluation on a scale of 1 (hardly applicable) to 5 (strongly applicable) with standard deviation. The course was evaluated by both occupational groups as being important to professional practice. The participants were more aware of each other in the simulation than they were in normal practice.

The spoken responses at the end of the teaching sequence gave no indication that immediate changes to the course design were needed. Both days were conducted in an identical manner. The evaluations are presented as the mean value of the five-point scale for all participants with standard deviation (see figure 2 (Fig. 2)). The actions of the nursing students were rated in terms of comprehensibility with 4.4 (±SD 0.67) by the nursing students and by the medical students with 3.75 (±SD 0.83). The fifth-year medical students rated the comprehensibility of their actions with 4.1 (±SD 0.44) and the nursing students rated them very nearly the same with 4 (±SD 0.71). Team coherence between physician and nursing staff was rated with 3.15 (±SD 1.19) by the nursing students and 3.5 (±SD 1.22) by the fifth-year medical students. During the simulation, the results here were 4.5 (±SD 0.59) for the nursing students and 4.38 (±SD 0.69) for the fifth-year medical students. Simulation as a teaching method with the goal of making the actions of the other profession understandable was evaluated by the nursing students with 4.15 (±SD 0.79) and the fifth-year medical students with 4.5 (± SD 1.22). The question about the usefulness of the acquired knowledge in terms of professional practice was rated with 4.05 (SD ±0.82) by the nursing students and with 4.00 (±SD 1.23) by the fifth-year medical students. The questionnaires were not analyzed according to gender due to the small sample size.

Excerpts of the responses to the open-ended question about growth of personal knowledge are presented in attachment: text box 5 (in total 16 nursing and six medical students). There were no negative statements. The term “communication” is mentioned more than once, as is the chance to do practical exercises. Contact with the other professional groups was described as being “great”.

## 4. Discussion

The aim of the authors was to develop a course concept that allowed participants to practice, in as real a situation as possible, their later role in a medical team. Using simulated scenarios as a teaching method can enable successful exchange of information between the team members and increase the quality of patient care [12]. As a form of training, simulation offers a safer learning context than the on-the-job reality of a hospital ward. In the debriefing sessions value was placed on having teams identify areas for improvement before hearing criticism from the passive observers. Self-correction by the teams under guidance is supposed to lead to better performance in subsequent situations [13]. No structured needs analysis in respect to nursing or medical students was carried out prior to the course. This is called for in the literature to provide effective training [14]. Despite this, the seminar was praised in the written evaluations as being very worthwhile and, as such, appears to have met the needs of the participants. When designing the course, we ascertained that no uniform catalogue of competencies exists for both professional groups. For this reason, the competencies identified in crisis management were drawn upon and included in the training center’s catalogue. To provide effective and safe patient care, the core elements of communication and being able to change perspectives are named. In particular, aspects such as closed communication loops can be practiced in simulated situations and are factors influencing patient safety [15]. Practicing these rules and routines formed the most important knowledge gain for most of the students.

The sense of being a team, a success factor for teamwork [16], was rated somewhat higher for the simulation than for actual routine work. For nursing students this difference was stronger than for the fifth-year medical students. According to our interpretation, the nursing students were more likely to view themselves as assistants in the hospital emergency. During the simulation the trainers were able to shift this perception since it was explicitly stated in advance how import active cooperation is from all those involved when it comes to successfully mastering a situation. The simulated training appears to be a meaningful intervention in this regard.

The fifth-year medical students were less able to understand the actions of the nursing students during the simulated scenarios. According to our interpretation, this was due to the fact that the medical students were under more pressure during the simulation and had to make final decisions about treating the patient. The ability to switch perspectives and view things from the vantage point of the other team member is also a factor for successful teamwork [17]. The training course can attempt to place more focus on understanding the actions taken by other professional groups.

The question regarding whether or not emergency patient care is improved as a result of knowing about crisis management is still open. Proof is difficult to find in the complex environment of a hospital. During the simulation, criteria from aviation such as the NOTECHS system [18], could serve as objective criteria for evaluating a team’s response. Since this project was primarily focused on the subjective evaluation of the participants and acceptance of the teaching method, any objective evaluation methods would have exceeded the project’s cope and the available resources, particularly in terms of staffing.

## 5. Conclusion

A one-day seminar cannot eliminate the multi-factor potential for conflict in hospital settings. Yet, this short training sequence has shown that practicing later professional roles during formal education is met with a high level of acceptance. From our point of view, clear formulation of the competency-based objectives in advance with a priority on non-technical skills is necessary. The trainers were aware, independently of the evaluation, that the conflict between physicians and nurses was constructively discussed by the student participants even in the breaks. A transparent and standard format for holding follow-up discussions helps put what has been personally experienced into an objective light. These debriefing sessions seldom occur in hospital settings so that the various perspectives and perceptions cannot be acknowledged. A joint training session at the beginning of the medical career appears to make sense since during this period a large part of the hospital-relevant socialization takes place. What remains questionable is whether or not the participants have the chance to integrate the non-technical skills they do learn into their routine clinical work. A longitudinal evaluation of interprofessional education in this area was not intended to be within the project’s scope. Further academic studies verifying a sustained improvement in the quality of patient care as a result of interprofessional training are desirable. Whether or not objective evaluation factors regarding non-technical skills come to be included in medicine, and if so how, will remain of interest.

## Notes

^1^ FIPPS is a German acronym for “Error management, Emergency care, Interdisciplinary, Professional per Simulation”

## Competing interests

The authors declare that they have no competing interests.

## Supplementary Material

Text boxes
