# Supplementary material for: Simulating a patient's fall as a means to improve routine communication: Joint training for nursing and fifth-year medical students
Source: GMS J Med Educ. 2016 Apr 29;33(2):Doc19. doi: 10.3205/zma001018 (PMC4895856; doi:10.3205/zma001018)
Supplement: Text boxes [file JME-33-19-s-001.pdf]

Text boxes

1. Competency objectives for the interprofessional simulation training involving a patient's fall in a hospital room

**Methodological competence**	**Social skills**	**Personal skills**	**Awareness of one's own responsibilities**
Ability to analyze<br>Symptoms are assessed pathophysiologically and treated appropriately	Empathy<br>The patient is treated as a person: procedures are explained and fears are taken seriously	Ability to handle stress<br>Despite pressure (expectations), the participant continues to keep working.	Participants make decisions within their sphere of responsibility and accept the consequences (e.g. the patient remains on the ward and undergoes further examination).
Focus on target group<br>Relevant information is passed on to team members and all others providing patient care.	Communication<br>Closed communication loop (instructions and commands are repeated), co-workers are addressed in a clear, unmistakable manner (by name).	Flexibility<br>New information is included in the diagnostic findings and applied to therapy.	
Organization<br>Priorities are set, tasks are planned in advance with an eye to the future.	Ability to handle conflict<br>Different ideas about therapy are mentioned and prioritized in the team.	Creativity<br>Pragmatic solutions are sought and implemented by the team.	
Problem solving<br>After making the suspected, symptom-based diagnosis, the correct plan of action is initiated.	Team skills<br>Tasks are assigned; the participants understand they are all part of a larger group.	Independence<br>The participants are in a position to make decisions and assume responsibility.	
	Ability to convince<br>The team is informed in a timely manner about all things under consideration and can easily understand the actions that are taken, and why.		

1. Simulated scenarios – “A patient falls”

**Clinical picture**	**Initial situation**	**Vital functions / pain according to visual analogue scale**	**Underlying patho-physiology**	**Other related care**
Femoral neck fracture and head wound	The patient is lying by the bed groaning. Small lacerations on the head. The patient’s leg appears shortened and is externally rotated.	Consciousness: normal<br>Breathing: normal<br>Pulse:<br>RR:<br>Pain: VAS 7	The patient has stumbled on the telephone cord.	Assistance,<br>Suspected diagnosis,<br>Analgesics,<br>X-ray imaging
Dementia	The patient has fallen on the way to the toilet. The neighboring patient was woken by the noise and calls for the nurse. The patient sits next to the bed and cannot provide an explanation.	Consciousness: fully unoriented (as described)<br>Pulse: 90/min, regular,<br>RR: 160/70mmHG<br>Pain: VAS 0	The patient was looking for the bathroom and has tripped on a cable.	Assistance,<br>Determine if any injuries are present,<br>If needed, x-ray imaging to rule out injury
Hypoglycemia	The patient is lying next to the bed, the neighbor calls for help and reports that the patient has been very confused for 30 minutes already.	Consciousness: difficult to wake, lack of orientation<br>Pulse: 130/min, regular,<br>RR: 90/40 mmHG<br>Pain: cannot be assessed	The patient was admitted for elective knee surgery and is required to fast prior to the OP; patient did inject self with insulin.	Assistance,<br>Diagnosis of hypoglycemia,<br>Glucose i.v.,<br>Examination for injuries
Getting up too soon after spinal block	Patient underwent outpatient surgery for an abscess and has gotten up too early on his own; the patient has collapsed, slipped against the bed, and is now sitting on the floor complaining since he had been promised he could go home at noon.	Consciousness: normal<br>Pulse: 90/Min regular,<br>RR: 160/70mmHG<br>Freq.: 100ppm RR 90/70mmHG<br>Pain: VAS 0	Spinal anesthesia has not fully worn off. Insufficient control of extremities for walking without assistance.	Assistance,<br>Help returning patient to bed,<br>Re-evaluation of pain when spinal block has worn off.
Anaphylaxis	Patient is lying on the floor by the window and gasping for air.	Consciousness: normal<br>Pulse: 110/Min regular,<br>RR: 90/40mmHg<br>Pain: VAS 2, ubiquitous spastic breath sounds.	The patient has already been given perioperative antibiotics on the ward and has gone into allergic shock.	Assistance,<br>Discontinuation of antibiotic therapy,<br>Diagnosis,<br>Diff. diagnosis,<br>Pharmacotherapy, Transfer to ICU

2. Guidelines for crisis management–Crisis Resource Management [http://www.nklm.de]

1. Know the environment.<br>2. Anticipate and plan.<br>3. Call for help early.<br>4. Exercise leadership and followership.<br>5. Distribute the workload (10 for 10 principle).<br>6. Mobilize all available resources.<br>7. Communicate effectively.<br>8. Use all available information.<br>9. Prevent and manage fixation errors.<br>10. Cross (double) check.<br>11. Use cognitive aids.<br>12. Re-evaluate repeatedly.<br>13. Use good teamwork.<br>14. Allocate attention wisely.<br>15. Set priorities dynamically.

3. Dash concept [6]

Core elements of debriefing, Boston Center of Medical Education
1. Instructor set the stage for an engaging learning experience.<br>2. Instructor maintained an engaging context for learning.<br>3. Debriefing was structured in an organized way.<br>4. The instructor provoked in-depth discussions.<br>5. The instructor identified what I did poorly or well, and why.<br>6. Instructor helped me see how to improve or sustain good performance.

4. Open-ended responses of the evaluation – My personal growth in knowledge

Nursing students:
- The simulation was very realistic, helped lead to an understanding of physicians' actions, and strengthened my self-confidence in emergency situations.<br>- Communication is very important between the two professional groups. Coordinating and cooperating makes work easier for us and the patient is better cared for.<br>- The simulation was very close to what happens in practice and strengthened my understanding of why doctors do what they do, while building my own confidence about my role in emergencies.<br>- Communicating information clearly, not just to think, but to share my opinion more often.<br>- Handling stress, when and how to communicate, how to approach situations.
Fifth-year medical students:<br>- Great experience, an advantage in all respects for my future work.<br>- I noticed how essential communication is and that I don't have much professional experience; it was great to have a chance to have contact with people from nursing.<br>- Without communication there is no effective teamwork.
